# Stress Distribution and Fracture Toughness of Underground Reinforced Plastic Pipe Composite

**DOI:** 10.3390/polym13132194

**Published:** 2021-06-30

**Authors:** Mohammed Y. Abdellah, Rami Alfattani, Ibrahim A. Alnaser, G. T. Abdel-Jaber

**Affiliations:** 1 Mechanical Engineering Department, College of Engineering and Islamic Architecture, Umm Al-Qura University, Makkah 21421, Saudi Arabia; rafattni@uqu.edu.sa; 2 Mechanical Engineering Department, Faculty of Engineering, South Valley University, Qena 83521, Egypt; gtag2000@yahoo.com; 3 Mechanical Engineering Department, King Saud University, Riyadh 11421, Saudi Arabia

**Keywords:** pipelines, glass fiber-composite pipes, surface release energy, cylinder

## Abstract

Reinforced composite materials have many applications in the aerospace, marine, and petroleum industries. Glass fiber-reinforced pipes are of considerable importance as pressurized vessels, infrastructure materials, and petroleum wastewater pipelines. The stress intensity factor due to through-thickness discontinuities is a major parameter in fracture mechanics to understand the failure mechanisms in glass fiber-composite pipes. The stress intensity factor is calculated for a composite cylinder subjected to internal pressure using the linear extended finite element method based on the law of energy release evaluation of surface damage. The analytical model needs two material properties; they are the tensile strength and the fracture toughness; therefore, a standard tensile test was carried out on a standard specimen taken from the pipe’s wall thickness. Moreover, the compact tension test specimen was manufactured from the pipe’s wall thickness to obtain the fracture toughness. The average tensile strength was measured as 21.5 MPa with a standard deviation of 5.59 MPa, moreover, the average Young’s modulus was measured as 32.75 GPa with a standard deviation of 6.64 GPa. The fracture toughness was measured as 2322 ($MPa\;\sqrt{m}$) with a standard deviation of 142.5 ($MPa\;\sqrt{m}$), whereas the average surface release energy ($G_{IC}$) was 153.6 kJ/m^2^ with a standard deviation of 22.53 kJ/m^2^. A valuable design equation was extracted from the finite element model to measure the effect of cracks on the hoop stress of the cylinder wall thickness based on a nonlinear model. Moreover, an acceptable equation was used to calculate the correction and shape factor of a cylinder with movable and unmovable through-thickness cracks. This study provides useful tools and guidance for the design and analysis of composite cylinders.

## 1. Introduction

Glass fiber-composite pipes play a dominant role in the infrastructure of any petroleum field and are an attractive alternative to metallic pipes in many industries. These pipes consist of glass fiber and thermosetting resin, and in many cases, quartz particles are added to form a sandwich composite material, which is generally made using the filament winding technique. Composite pipes have many applications, including pressure pipelines and water transportation over and underground [1,2,3]. The degradation of the fracture and mechanical properties of GRP pipes after immersing in a corrosive medium (solution of sodium chloride in water) was reported [2,4]. This degradation was attributed to the corrosion occurrence utilizing a corrosive medium for only composite pipes.

Wastewater influences the tensile and flexural strength and fracture toughness of reinforced-composite pipes in the petroleum field [2]. The decrease in these properties compromises the safety of pressurized pipes, and the existence of small cracks throughout the pipes’ wall thickness may cause abrupt failures. Rafiee and Ghorbanhosseini [5] investigated the creep and long-term properties of composite pipes under internal pressure and transverse compression loading. Other works on compression creep were carried out by Farshad and Necola [5,6], but the pipe material was conditioned in water at ambient temperature before testing, and it was found that the properties were negatively affected. In addition, water has a detrimental effect on the flexural strength of glass fiber-reinforced pipes depending on the air conditions [7]. Composite pipes are distinguished by their low cost and corrosion resistance, but the decrease in strength is a significant problem; Miao et al. [8], for example, coated a composite with basalt fiber and epoxy resin, achieving good results. The hygrothermal effect and bending properties of composite pipes modified with graphene nanoplatelets to increase their service life were studied by Firouzsalari et al. [9]. Dong [10] studied the failure of composite pipes and measured residual stresses using a finite element model. Moreover, the effects of the layup and fiber orientation were studied. Another finite element study [11,12] investigated the effect of thickness, winding direction, and the number of fabric plies of composite cylinders under internal pressure on the characteristic mechanical properties using the analytical finite element method. The hoop stress distribution or the circumferential thickness tensile strength was calculated numerically and experimentally using a split-disk test method [13], which was based on a progressive damage model through finite element analysis of a cracked disk. A nondestructive analytical model based on vibration modal analysis was extracted by Abdellah et al. [14] to predict the nominal strength and fracture properties of glass fiber-composite laminates. A direct link between the specimen densities and the dimensional accuracy with orientation was observed [15]. Moreover, the angular orientation led to significant anisotropic behavior in terms of fracture toughness of single edge notch samples. The effect of seawater aging and curing on similar polymer-composite cylinders used as the marine structure was evaluated [16]. Completely cured composite cylinders had significant mechanical properties compared with partially cured ones. Besides, aging in seawater for a certain time resulted in enhancing both hoop strength and stiffness, which again was not critical for radial strength. Similarly, the hardness and density of the same cylinders under seawater exposure were investigated and were found to be changed with the effect of seawater presence [17]. Moreover, the effect of seawater on steel pipes that were rehabilitated with layers of fiberglass and epoxy was studied [18]. The hoop strength of pipes being repaired before and after immersion increased, indicating the need for a greater thickness of composite repair for complete rehabilitation.

The mechanical properties of composite cylinder material were measured using standard tensile strength [19] to be implemented in a finite element analysis which was used to calculate the static performance of the composite cylinder. Through the numerical study, the stress analysis and Von Mises stress were obtained. Composite cylinder pipes which were reinforced by E-glass and T300/934 were investigated [20] under pressurized medium to obtain the hoop and radial stress induced through the cylinder wall thickness. The composite cylinder with particular characteristics was used in compressed hydrogen storage applications [21]. For more focus on fracture mechanics, the notch effect on cylinders under internal pressure was considered [22]. The failure modes of the composite cylinder were investigated not only under the effect of internal pressure but also under the effect of low-velocity impact [23] using the layer-wise theory through progressive damage model. Delamination and bad damage were observed in composite pipes under static and impact load [24]. Rapid damage was in the critical load for a drop weight impact of a composite tube reinforced by fibers, therefore higher incident energy was revealed [25]. Glass/carbon functionally graded filament wound composite pipes under low-velocity impact and internal pressure were studied by Gemi et al. [26]. Delamination was observed on the outer surfaces of the pipes, moreover matrix cracking. Valuable finite element analysis was extracted to predict the mechanical properties and behaviors of the composite cylinder under pressure vessels, it was shown that high-density polyethylene (HDPE) was very attractive and had a good bearing and high-stress capacity, moreover, it was recommended that FEM was a good tool for simulating the pressurized composite cylinder [27]. Carbon-reinforced epoxy composite pipes were numerically and experimentally investigated under an external pressure, finite element model and an evaluation damage model was developed. Buckling and in-plane shear modes with delamination were observed [28].

The composite layup of composite pipes influenced their mechanical properties, a progressive damage model [29], was derived to predict the failure behavior of composite laminates with different layups and stacking sequences. The investigation used progressive continuum mechanics and the experimental results gave a robust technique to understanding the carbon fiber reinforced pipes failure mechanisms.

The flexural stiffness of thick-walled composite pipes was experimentally studied by Geuchy and Hoa [30], it was revealed the relation between experimental and theoretical results, moreover, it was given a great difference between the strength of material concept and elasticity concepts.

The relationship between the structural properties of the shell and the end bearing capacity (UBC) on fiberglass reinforced mortar tubes (GRP) was simulated [31]. It was observed that the UBC was in a growing pipeline with an increase in the proportion of the size of the fibers and the proportion of the volume of the spirally wound layer, but UBC increased with the layers of tubules.

On the other hand, the effect of thermal aging on the compression behavior of interlocking polymer network composites reinforced with fiberglass was discussed. The strength of these pipes decreased slightly with increasing temperature and concerning the corresponding aging period. Besides, the thermal conductivity of the glass fabric composite epoxy laminates was enhanced using hetero-structured BNN-30@BNNS fillers, the fillers accelerated the heat flow transfer [32].

A comparison between the carbon and energy footprint of nonmetallic composite pipes in onshore oil and gas flowlines was investigated using Life Cycle Assessment (LCA) analysis [33]. It was reported that a stronger and high-strength fiber, such as carbon fiber, gave better weight safety for pipes.

Not only do polymer matrix composites have attractive and competitive roles in aerospace industries but also lately they have been inserted into the transmission of electromagnetic waves and protective antenna [34]. These types of polymers, matrix reinforced with other advanced fibers such as PBO fibers [35], but these types of fiber may need a special coating to increase adhesiveness at the interfacial interface by the cyanate ester polymer [36]. Another fiber proposed for use in this advanced process is dopamine/POSS functionalized Kevlar cloth may be more compatible for wave transmission [37].

### The Novelty and Objectives of the Present Study

In the present study, the stress intensity factor of a composite cylinder with a crack was studied, based on an extended finite element model, which requires two parameters: the traction separation at which the crack propagates and the damage evaluation, which requires knowledge of the surface release energy. Therefore, a tensile test of the cylinder thickness was carried out and compact tension test specimens were performed.

Therefore, the main goals of the present study are as follows:

- Measure the stress intensity factor of the cracked cylinder using the extended finite element model.
- Extract an analytical model to predict the hoop stress of the cracked composite cylinder which is a modification for the classical Lame’s Equation (2) [38], for any shape, not for an elliptical crack as in Newman [39].

The paper is organized as follows: mathematical theory, the finite element model, results and discussion, conclusion.

## 2. Mathematical Model

The mode I stress intensity factor $K_{I}$ at the free surface of crack can be calculated by Newman [39] as follows:

(1) $$K_{I}=\sigma_{H}\sqrt{\pi \frac{a}{Q}}\times f\left(\frac{a}{t},\;\frac{R}{t},\;\frac{a}{c}\right)$$

where $\sigma_{H}$ is the hoop stress through the cylinder wall thickness (t), (a) is the crack length, (b) is the crack width through the thickness (rectangular or straight crack), Q is the shape factor, and R is the cylinder inner radius. The hoop stress for an unnotched thin cylinder (t/d < 20) or through-thickness unopen crack can be measured using Equation (2) [40]:

(2) $$\sigma_{H}=\frac{P_{i}d}{2t}$$

**Figure 1:**
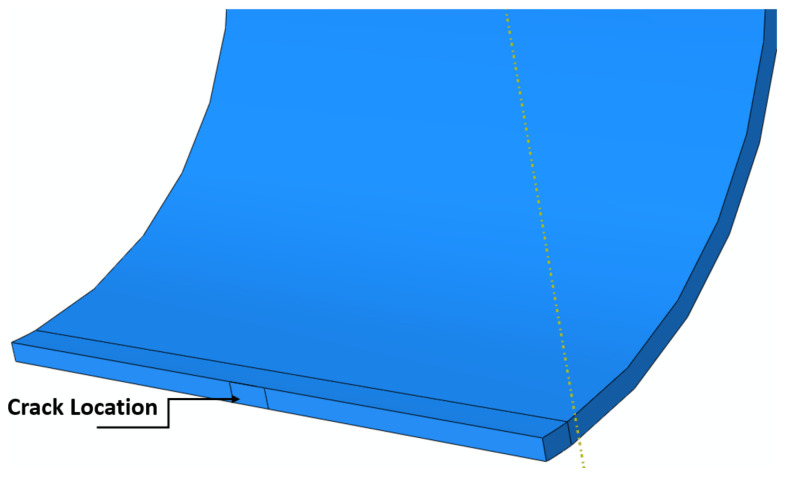
Crack location through the thickness.

The longitudinal stress $\sigma_{L}$ is half of the hoop stress, assuming closed-end cylinders. For open through-thickness cracks with straight lines (rectangular cracks in Figure 1), the hoop stress would be dependent on the crack length; therefore, the FEM was implemented and the stress intensity equation by Newman was modified as follows:

(3) $$K_{I}=\sigma_{H}\sqrt{\pi a}\times \frac{f\left(\frac{a}{t},\;\frac{R}{t},\;\frac{a}{c}\right)}{\sqrt{Q}}=\sigma_{H}\sqrt{\pi a}\times Y$$

Factor Y is the total effective parameter correction factor or influence function, which can be measured as follows:

(4) $$Y=\frac{(K_{I})FEM}{\sigma_{H}\sqrt{\pi a}}$$

## 3. Extended Finite Element Model (XFEM)

Belytschko and Black [41] recently developed the XFEM. The main idea of the XFEM is based on the work of Melenk and Babuska [42] who used the concept of partition of the finite element unity and enrichment function. XFEM is distinguished because the mesh does not have to be updated to follow the crack path [43], and therefore, the need to mesh and re-mesh the complex discontinuity surfaces is reduced. The analysis of a fracture can be performed with considerable numerical accuracy as the crack propagates around the crack tip without re-meshing and refinement; for a complete description, see [44].

**Figure 2:**
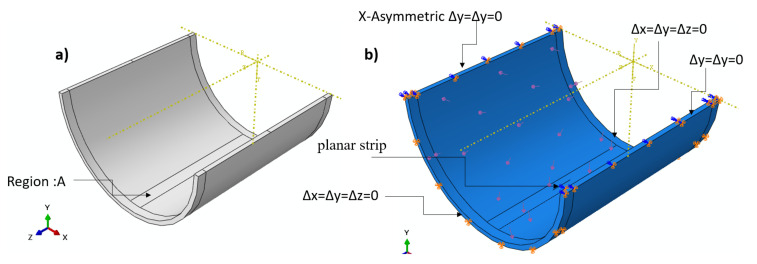
FE (**a**) solid domain, (**b**) boundary condition domain.

**Figure 3:**
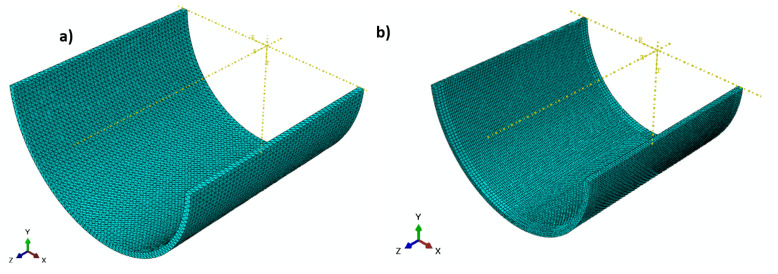
FE mesh (**a**) tetrahedron shape (un-notched), (**b**) hexagonal shape (notched).

The 3D nonlinear elastic FEM is based on the basic extended fracture method. A cylindrical domain (520.7 mm diameter × 500 mm) was created as a solid part (see Figure 2 a). A straight rectangular planar strip (a × 13 mm) was implemented. Crack lengths (a) of 10, 20, 30, 40, 50, and 60 mm were investigated. The crack was movable for the calculation of the measured hoop stress through-thickness and crack propagation behavior under an internal pressure $P_{i}=15\;MPa$. To calculate the stress intensity factor using the linear elastic extended finite element method, the crack was fixed and kept unmovable. The material used for the composite cylinder was based on DN 500 and SN 1000 [45]. The maximum principal stress corresponded to the unnotched nominal strength, which was measured as 30 MPa. The damage evaluation criterion is the maximum fracture energy (185 kJ/m^2^) and the independent mixed-mode that is applicable. A 10-node quadratic tetrahedron shape (47288) C3D10 element type with 10 global sizes was used for an unnotched cylinder (see Figure 3a). Additionally, 38412 C3D8R elements with an approximately hexagonal shape with a global size of 10 and 5 for region B were used in the domain, as shown in Figure 3b. The displacement boundary condition technique was applied at both ends of the cylinder cracked domain (see Figure 2b) for internal pressure.

## 4. Experimental Work

The previous numerical model required the mechanical properties of the cylinder wall thickness; therefore, a tension test was carried out to obtain the composite Young’s modulus and tensile strength. The fracture toughness and related longitudinal surface energy release for fiber tension were needed to evaluate the damage model implemented in the FEM.

### 4.1. Material Description

**Figure 4:**
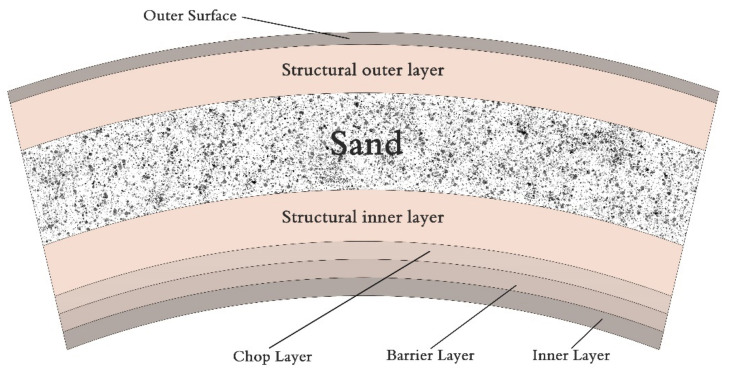
Schematic drawing of GRP cross-section.

The present study used glass fiber-reinforced polymer pipes with a heterogeneous structure consisting of random matt, roving, unsaturated polyester resin, and sand according to the values displayed in Figure 4 and listed in Table 1. An unsaturated polyester resin brings environmental and chemical resistance, it also bonds the fiber in the pipe structure. Unsaturated polyesters are less expensive than other resins used in GRP pipe manufacturing but provide a slight strength and chemical resistance. It is often an economical choice for a less demanding, low-pressure service.

The glass fiber composite pipes were manufactured using the common filament winding technology. The GRP has a complex structure, inner and outer surface layers. Then the barrier and chop layer end by the structured layers in the outer and inner surfaces, where inbetween quartz sand was placed. For a complete description of the manufacturing process return to [18]. The composite material mainly depends on the fiber geometry, lay-up thickness, fiber pretension, and the quality of the manufacturing process.

**Figure 5:**
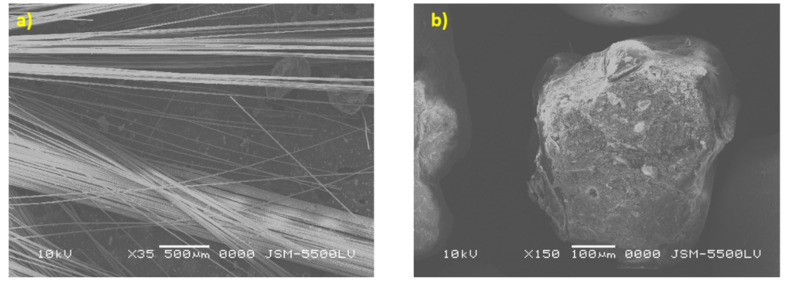
GRP constitutive contents (**a**) fiber shape, (**b**) quartz sand shape and size.

These constituent compositions were obtained using the ignition removal technique, according to the standard ASTM D3171-99 [15]. These types of pipes are used in pipelines for chemical wastewater used in the petroleum field. The fiber shape and size are illustrated in Figure 5a, and the grains of quartz sand and shapes are shown in Figure 5b. The elastic properties of the glass fiber composite pipes (GRP) are listed in Table 2. The equivalent Young’s modulus is 35.15 GPa, which is calculated based on Reference [46] and experimental tensile data.

Although the pipes are taken ex situ, and they were in service for a period of time, this action would not affect the results of the mathematical model because the model depends on the as-received input data, and the validation was carried out on the as-received experimental data of the material of the in situ pipe.

### 4.2. Tension Test

**Figure 6:**
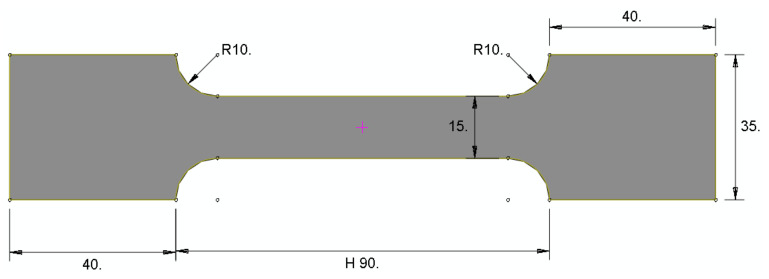
Tensile test specimen geometry.

Tension tests were performed on specimens manufactured from glass fiber-reinforced polymer pipe wall thicknesses (DN = 520.7 mm and 13 mm thickness). Four specimens were tested. These four specimens were cut from the wall of the composite pipes using a diamond coated disk to obtain the final dimension which is shown in Figure 6. A curvature of 10 mm radius was created at the corner of the specimen to distribute uniformly the stress through the cross-section of the specimens. The width of the specimen was selected to be as minimal as possible to decrease the large curvature. These tests were performed according to ASTM D3039 [47] using a universal testing machine (machine model WDW-100) with a load capacity of 20 kN and a controlled speed of 2 mm/min. Figure 6 shows the standard specimen geometry for tension.

### 4.3. Fracture Toughness Test

**Figure 7:**
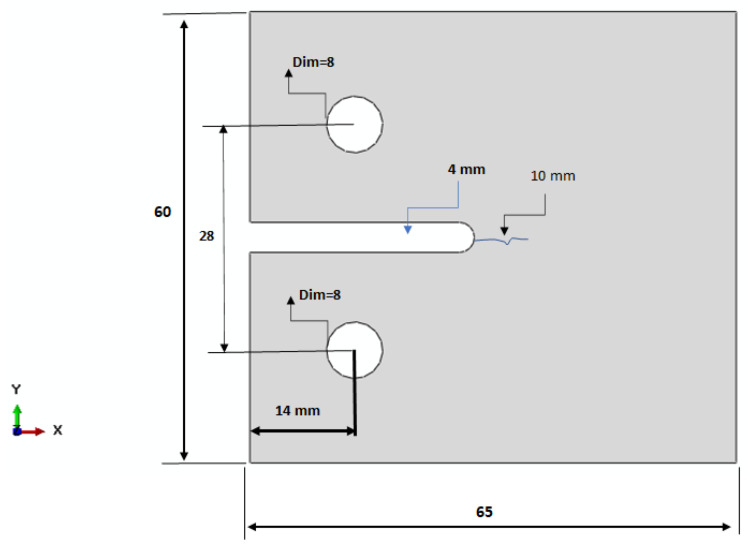
CT test specimen geometry.

The fracture toughness of such materials is more important, as the occurrence of any crack will cause the absorption of water through the GRP pipe sandwich. This can cause harmful corrosion, which may subsequently result in degradation. Therefore, fracture toughness is an attractive parameter to be measured. The corresponding surface release energy G_IC_ was needed in the previous derived analytical model. One type of fracture toughness test on the specimens was used according to ASTM standard D 5045 [48]. The crack resistance must be measured so as to be stopped at the onset of degradation and obtain satisfactory fracture toughness results. The three compact tension specimens were machined using a diamond-coated disk from the wall material of glass fiber reinforced pipes (GRP pipes) according to the dimensions given in ASTM D 5045, as shown in Figure 7. The loading holes were then drilled using tungsten carbide drills while clamping the specimen between two sacrificial plates of the same material to prevent damage and delamination. A 10 mm starter crack was created using a fine razor blade. The crack face was given a scaled mark. The thickness of the specimen was 13 mm.

## 5. Results and Discussion

**Figure 8:**
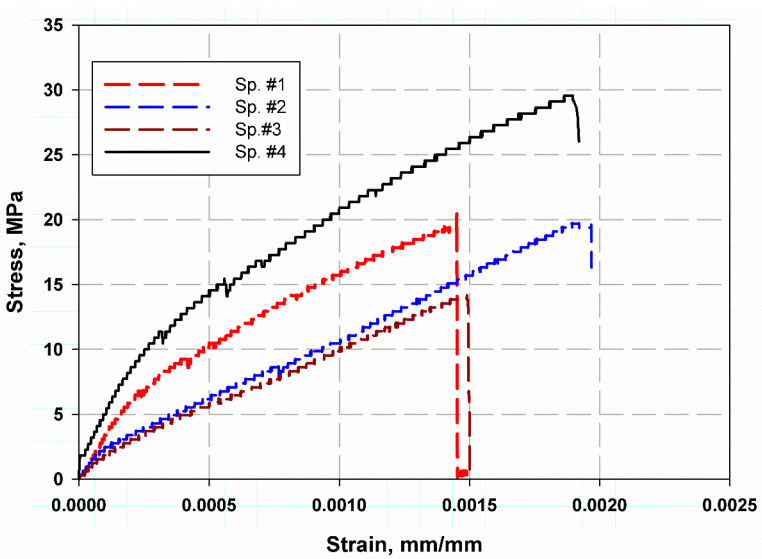
Stress–strain curve for the tension of GRP pipe material.

**Figure 9:**
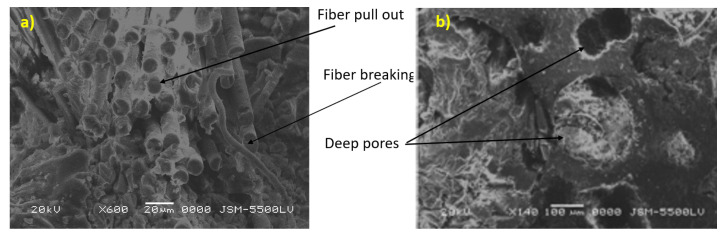
(**a**,**b**) SEM examination of fracture surface in the tension test specimen.

The tensile test results are shown in Figure 8, which shows that the curved section of the cylinder material had an average strength of 21.5 MPa with a standard deviation 5.59 MPa. The average Young’s modulus was 32.75 GPa with a standard deviation 6.64 GPa. The curve was not smooth and had a stepwise behavior, attributed to fiber bridging (pull-out), fiber failure as shown in the SEM image in Figure 9a. The fracture was nearly ductile as pores in the matrix are observed in Figure 9b. In a focus observation of the curve behavior, all specimens nearly had the closest elongation, the curve trend was not entirely linear, and there was some plasticity or hardening because the composite material is considered a quasi-brittle material [49,50,51,52], which was distinguished by a moderate plastic zone ahead of the crack tip. There were also two specimens with black and red colours similar to two knees (two lowering points), which corresponded to the first and second fiber failure in the transverse direction [53]. Redistribution of stress between longitudinal fibers and matrix led to a drastic nonlinear increment [54] in the tensile stresses with a tensile modulus lower than that for the initial linear portion. The final failure was catastrophic without any yielding.

**Figure 10:**
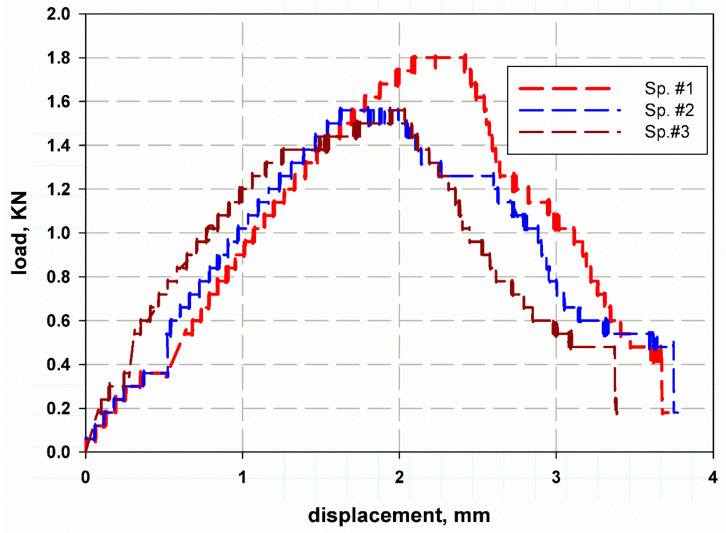
Load–displacement curve of different compact tension specimens.

The load–displacement curve for a standard compact tension test specimen is needed to complete the specific failure mechanisms of the curved wall thickness of the cylinder to measure its fracture toughness. Crack growth was neither smooth nor continuous; instead, several crack jumps of a few millimetres each time were observed, as shown in Figure 10. The peak load, $p_{Q}$, which was 5% according to the standard ASTM D 5045, was determined [48]. This load corresponded to the maximum load capacity of the material before failure. The peak load values for the three specimens were inserted into Equation (5) to calculate the mode I fracture toughness ($K_{IC}$) values ($MPa\;\sqrt{m}$). According to the standard ASTM E399 [55], the critical stress intensity factor for a fracture load ($p_{Q}$) is given by

(5) $$K_{IC}=\frac{p_{Q}}{t\sqrt{w}}f\left(a/w\right)$$

where (t) is specimen thickness, mm; (w) is specimen width, mm; (a) is crack length, mm; ($p_{Q}$) is the load at 5% secant; and (f(a/w) is the shape correction factor:

(6) $$f\left(a/w\right)=\frac{2+a/w}{{\left(1-a/w\right)}^{1.5}}[0.886+4.64\left(a/w\right)-13.32{\left(a/w\right)}^{2}+14.72{\left(a/w\right)}^{3}-5.6{\left(a/w\right)}^{4}$$

The critical energy release rate of the laminate can be calculated from $K_{IC}$ as follows [56,57]:

(7) $$G_{IC}=J_{IC}=\frac{K_{IC}^{2}}{\psi }$$

where Ex, Ey, Gxy, and $\nu_{xy}$ are Young’s modulus in the (x) and (y) directions (see Figure 7), the shear modulus, and the Poisson’s ratio of the laminate, respectively, which are listed in Table 2. The equivalent young (ψ) is calculated as follows [46]:

(8) $$\psi =\frac{\sqrt{2E_{x}E_{y}}}{\sqrt{\sqrt{\frac{E_{y}}{E_{x}}}+\frac{E_{y}}{2G_{xy}}-\nu_{xy}}}$$

**Figure 11:**
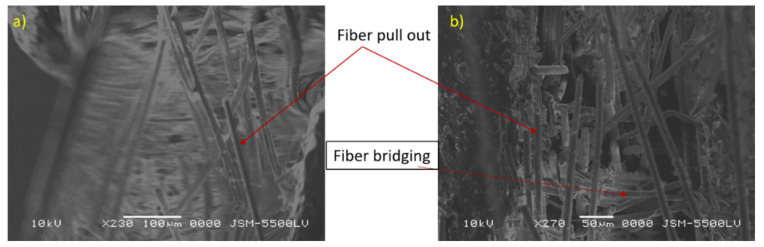
(**a**,**b**) SEM examination of fracture surface in compact tension specimen.

After substituting these reduction data, Young’s modulus was calculated to be 35.15 GPa, which is very close to the average experimental Young’s modulus (32.75 GPa). The optically observed failure crack length reached 22.5 mm. The average fracture toughness ($K_{IC}$) was measured as 2322 ($MPa\;\sqrt{m}$) with a standard deviation 142.5 ($MPa\;\sqrt{m}$). The average surface release energy ($G_{IC}$) is 153.6 kJ/m2 with a standard deviation 22.53 kJ/m^2^. It was observed that the fiber bridged the two faces of cracks, and the crack advanced straight through the precrack direction, owing to the high-stress intensity factor induced at the crack tip. The existence of cracks in the pipe surface or even macrocracks may be harmful and cause leakage of the petroleum waste fluids into the pipe core quartz particles, which can be dangerous [2]. Fiber pull out, fiber fracture, fiber bridging, and matrix cracking, are shown in Figure 11. The sand quartz has the major role in this trend of behavior. The sand core material in the cylinder is distinguished by high stiffness and brittleness, especially when combined by epoxy resin. The curvature of the specimen had a dominating role in the increase of the fracture toughness values. The curvature of the specimen redistributed the loading stress through the cross-section of the tested specimens.

The variation of the tensile strength values of each of the four specimens ranged from 30 MPa to 14 MPa for specimen number 1 and 4 respectively, this variation was due to an error in the specimen preparation, machine stiffness, misalignment error, the maximum variation percent were nearly 50% while minimum percent was 30%, this is because of the difficulty of curved surfaced deformation and machining. This range decreased in the case of the compact tension test specimens which were 1.8 kN to 1.5 kN (see Figure 10). This was because in the compact tension test the load decreased after reaching critical values.

This variation as previously mentioned is not a problem, and its effect on the mathematical model is slight because the model used the as-received input data, which were fed to the ABAQUS software.

### Mathematical Model Results

**Figure 12:**
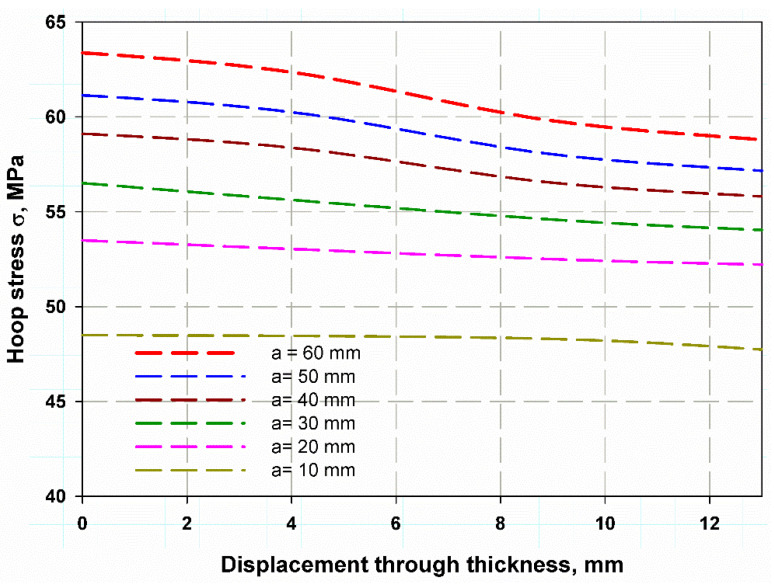
Hoop stress distribution across the thickness.

**Figure 13:**
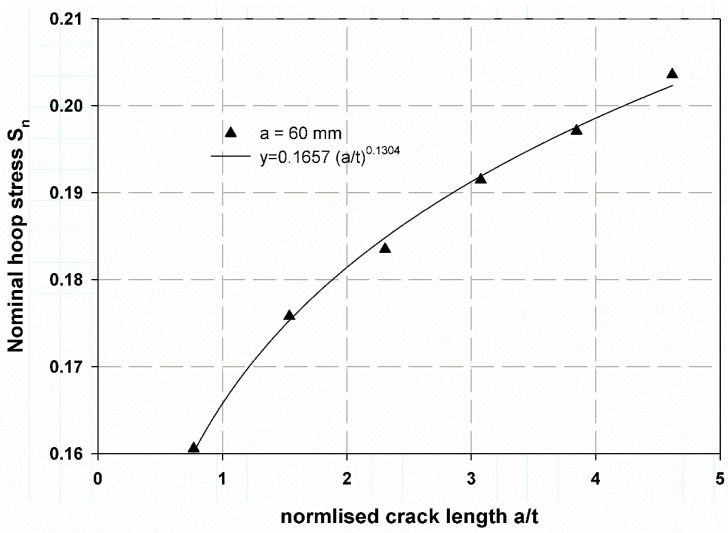
Nominal hoop stress versus the normalized crack length.

The stress distribution over the composite cylinder’s thickness is illustrated in Figure 12 for different through-thickness cracks. The hoop stress in the wall thickness of the cylinder was calculated using Lame’s Equation (2), [38]; it was determined to be 300.4 MPa for the uncracked cylinder. Cracks through the wall thickness are the major reason for the failure and damage of any cylinder with internal pressure or external pressure [39]. Therefore, the values of the hoop stress in the through-thickness movable crack are fitted in Figure 13 with the following logarithmic Equation:

(9) $$S_{n}=\frac{\sigma_{H-n}}{\sigma_{H-un}}=0.1657\times {\left(\frac{a}{t}\right)}^{0.1304}$$

**Figure 14:**
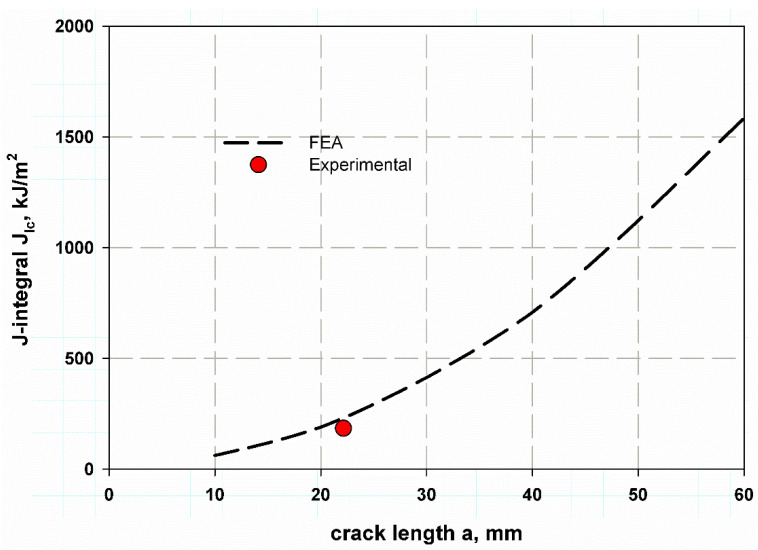
J-integral prediction using XFEM for through-thickness crack of the composite cylinder.

The fitting equation yields very close results from FEM, which can give a wide range of cylinders with different values of (a/t). This idea is similar to what was proposed by Newman [39]. This result may enable the extension of the data below and over the studied range of a crack length 10–60 mm. Figure 13 also can illustrate the effect of cylinder thickness on the stress distribution of Figure 12 also can illustrate the effect of cylinder thickness on the stress distribution through its cross-section. It was observed that increasing thickness meant decreasing the aspect ratio (a/t), which led to lower values of hoop stress. The stress distribution through the thickness was nearly close at a small thickness (see Figure 12). The average J-integral of the five contours is shown in Figure 14. It was predicted using the proposed XFEM for different crack lengths based on the wall thickness of the cylinder. The results of a crack length of 22.5 mm were very close to the compact tension test results. This trend of increasing J-integral with crack length increment is general in composite quasi-brittle materials [56]. The value of experimental fracture toughness is one obtained at critical crack length at the critical crack opening, while the J-integral value is a mathematical curve that passes through the propagation of the crack to infinity of crack length. If the path moves with the experimental value, which is one and constant for the material, then the prediction is considered acceptable. When I need to measure the R-curve of a material through the crack propagation, in this case, the need to obtain more values would be suitable and considered [58,59,60].

**Figure 15:**
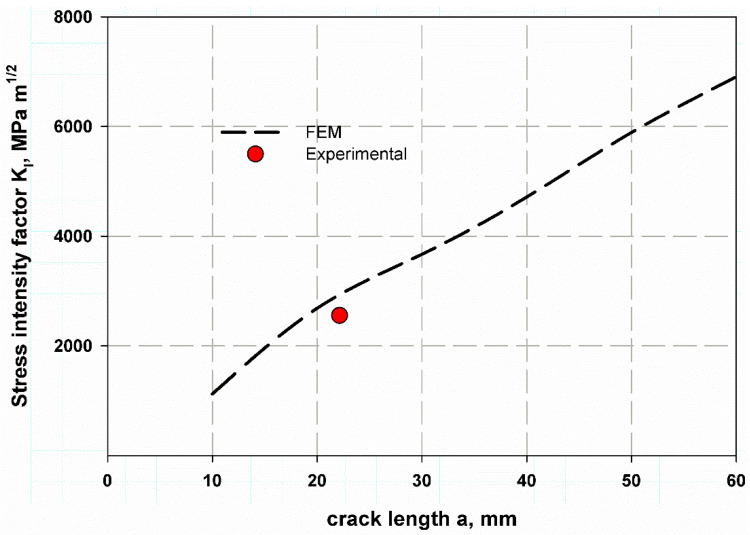
Stress intensity factor for thin-walled composite cylinder under internal pressure.

The stress intensity factor is shown in Figure 15, and the predicted values were very close to the experimental data reduction using Equation (5) at crack 22.5 mm. The trend again increases with crack increment, as it is assumed that the pipe length to infinity is very long and does not affect the analysis [39].

Table 3 shows the data predicted by Equation (10) of influence coefficients or correction factor based on the fitted data of the FEM for (a/t) ranges 0.2–0.8, and the cracks have rectilinear or square dimensions with movable (nonlinear step) and unmovable cracks (linear step). The large deviation between the data in Reference [61] and the present study is because the present model is for a straight plane crack (rectangular) and cylinder t/R = 0.045.

(10) $$Y=C\times \left(1-e^{-B\left(a/t\right)}\right)$$

where C and B are constantly obtained by the well-fitted curve.

**Figure 16:**
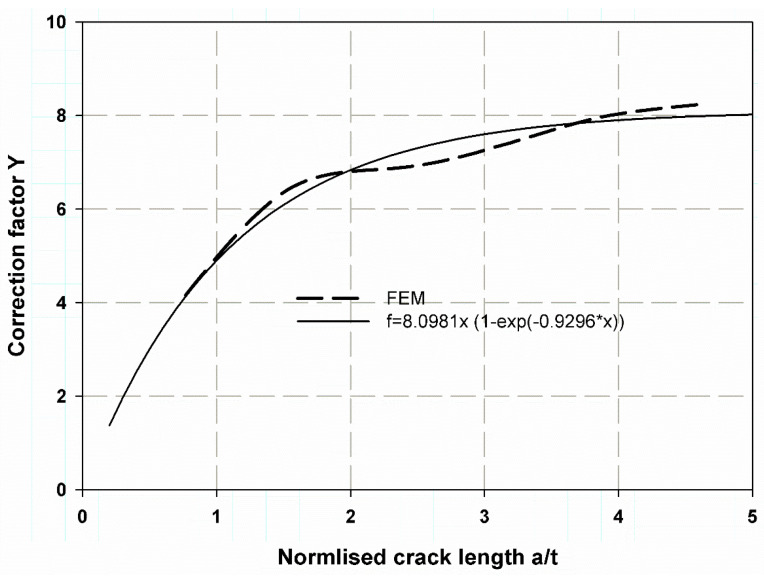
Correction factor for a movable crack through the thickness of the walled composite cylinder.

**Figure 17:**
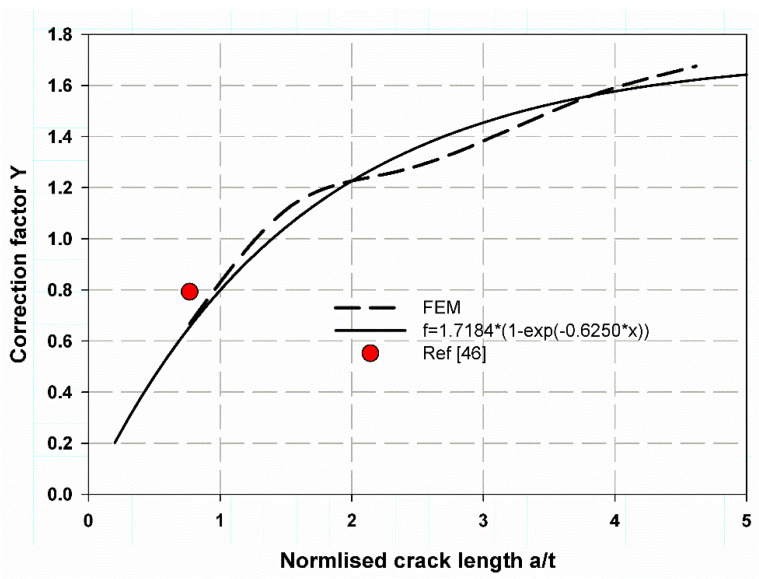
Correction factor for an unmovable crack through-thickness of walled composite cylinder.

**Figure 18:**
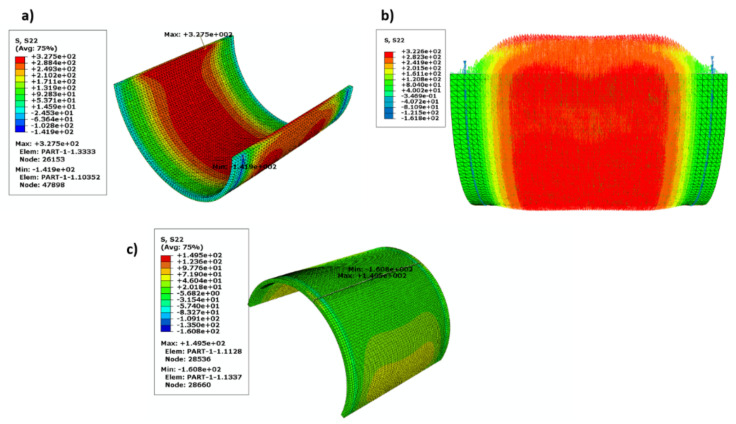
Hoop stress distribution (**a**) cylinder (**b**) cylinder with stress direction, (**c**) cracked cylinder.

Figure 16 shows the fitted data for the nonlinear FEM with a movable crack, which is a power exponential for a wide range of (a/t) 0.2:5. The results were acceptable and provided reasonable accuracy. This is clearly shown in Figure 17 for a linear FEM with an unmovable crack when compared with the work of [61]. The stress distribution over the wall thickness is shown in Figure 18 using the colour FE contour. The maximum hoop stress was induced through the uncracked specimen’s thickness (Figure 18a,b). However, it was reduced in the crack tips due to the stress concentration around the crack tips (Figure 15).

## 6. Conclusions

This work has successfully developed a new finite element model (FEM) for predicting the mechanical properties of cracked and neat composite cylinder materials for industrial applications. An expression was extracted to predict the stress intensity factor of a composite cylinder using an XFEM based on the enrichment function. The model yielded reasonably good results. Moreover, the hoop stress induced through the cylinder thickness was assessed for neat and cracked cylinders using XFEM. The stress distribution through the thickness of the cylinder due to internal pressure was demonstrated. It was found that the presence of through-thickness cracks decreases the average hoop stress values (61.2 to 48.5 MPa) through the cylinder’s thickness. The fracture toughness was about 2322 ($MPa\;\sqrt{m}$) with a standard deviation 142.5 ($MPa\;\sqrt{m}$), and its corresponding surface release energy was about 153.6 kJ/m^2^ with a standard deviation 22.53 kJ/m^2^ which were measured experimentally using a standard compact tension test specimen, which could be used as a fracture toughness standard measurement method for composite cylinder materials. The tensile test results were experimentally measured using standard tensile test specimens of the curved cylinder wall. Overall, taken together with the obtained results and our findings we can conclude that the proposed FEM would have a strong potential for predicting the mechanical properties of composite cylinder materials for industrial applications. Finally, the study is distinguished by fast procedures and few parameters. The model can be applied on many composite pipes reinforced by glass fiber with different shapes and geometry (mat or roving), just it is needed to know the mechanical characteristics of the tested material. Moreover, the numerical model enables the modification of the classical Lame’s Equation (2), and it is applicable for any shape, not just for an elliptical crack as in Newman.

## Figures and Tables

**Table 1:** Composition of GFR pipes [2].

Constituents	Average %	Viscosity/cp.25 °C	Thermal Deformation Temperature/°C	Tensile Strength/MPa	Specific Density (g/cm^3^)
Thermosetting unsaturated polyester (Matrix)	30.2%	400	70	65	1.12
Roving	11.8	-	-	3100–3400	2.5
Matt	13.5	-	-		
Sand	44.5	-	-	-	2.66

**Table 2:** Elastic properties of GRP [45].

Properties	E_1_ (GPa)	E_2_ (GPa)	ν_12_	G_12_ (GPa)	G_13_ (GPa)	G_23_ (GPa)
Value	100	9	0.3	3.2	3.2	4

**Table 3:** Influence coefficients Y for stress intensity factor with linear and nonlinear curve fitting.

a/t	Y (Nonlinear)	Ref. [61]	% Error	Y (Linear)
0.2	0.2019	0.197	2.43	1.3739
0.4	0.3801	0.31	18.44	2.5147
0.6	0.5374	0.458	14.77	3.462
0.8	0.6761	0.702	−3.83	4.2486

## Data Availability

The data presented in this study are available on request from the corresponding author.

## Nomenclature

$G_{IC}$	Critical surface release energy
$J_{IC}$	Critical J-integral
$K_{I}$	Mode I stress intensity factor
$K_{IC}^{2}$	Critical fracture toughness
$P_{i}$	Internal pressure
$S_{n}$	Nominal hoop stress=$\frac{\sigma_{H-n}}{\sigma_{H-un}}$
$p_{Q}$	5% secant load
$\sigma_{H}$	Hoop stress
$\sigma_{L}$	Longitudinal stress
**E_1_, E_2_**	Young’s modulus in 1 and 2 direction or X, Y
**G_12_, G_13_, G_23_**	Shear modulus in 1, 2, and 3 direction
**R**	Cylinder radius
**ν_12_**	Poisson’s ratio
C and B	Constants obtained by the well-fitted curve
Q	Shape factor
Y	Equivalent correction factor
a	Crack length
c, b	Constants form well-fitted curve
d	Cylinder diameter
$f\left(\frac{a}{t},\;\frac{R}{t},\;\frac{a}{c}\right)$	Correction factor for cracked cylinder
$f\left(a/w\right)$	Correction factor for compact tension test specimen
t	Cylinder thickness
w	Compact tension test specimen dimension
ψ	Equivalent Young’s modulus
